# Novel Insight into the Association between Balneotherapy and Functional State and Health Perception in Chronic Low Back Pain: A Cross-Sectional Study

**DOI:** 10.3390/jcm13175248

**Published:** 2024-09-05

**Authors:** Dragana Terzic Markovic, Sanja Kocic, Jovana Bradic, Aleksandra Jurisic-Skevin, Biljana Jakovljevic, Biljana Majstorovic, Zvonko Dimoski, Goran Stojanovic, Vesna Maksimovic, Katarina Pavlovic Jugovic, Marijana Dabic, Danijela Jezdimirovic, Sandra Zivanovic

**Affiliations:** 1 The College of Health Science, Academy of Applied Studies Belgrade, 11000 Belgrade, Serbia; biljana.jakovljevic@assb.edu.rs (B.J.); biljana.majstorovic@assb.edu.rs (B.M.); zvonko.dimoski@assb.edu.rs (Z.D.); goran.stojanovic@assb.edu.rs (G.S.); vesna.maksimovic@assb.edu.rs (V.M.); katarina.pavlovic.jugovic@assb.edu.rs (K.P.J.); marijana.dabic@assb.edu.rs (M.D.); danijela.jezdimirovic@assb.edu.rs (D.J.); 2 Department of Social Medicine, Faculty of Medical Sciences, University of Kragujevac, 34000 Kragujevac, Serbia; kocicsanja@yahoo.com; 3 Department of Pharmacy, Faculty of Medical Sciences, University of Kragujevac, 34000 Kragujevac, Serbia; 4 Center of Excellence for Redox Balance Research in Cardiovascular and Metabolic Disorders, 34000 Kragujevac, Serbia; 5 Department of Physical Medicine and Rehabilitation, Faculty of Medical Sciences, University of Kragujevac, 34000 Kragujevac, Serbia; jurisic-skevin.aleksandra@medf.kg.ac.rs; 6 Department of Physical Medicine and Rehabilitation, University Clinical Center of Kragujevac, 34000 Kragujevac, Serbia; 7 Department of Natural Sciences, Faculty of Hotel Management and Tourism, University of Kragujevac, 36210 Vrnjačka Banja, Serbia; zivanovicsandra@hotmail.com; 8 Faculty of Medical Sciences, University of Kragujevac, 34000 Kragujevac, Serbia

**Keywords:** chronic low back pain, balneotherapy, functionality, life quality, pain

## Abstract

**Background:** Epidemiological data indicate that low back pain (LBP) affected 619 million people globally in 2020, representing a significant health and economic burden. Additionally, chronic LBP (cLBP) strongly impairs quality of life and leads to disability and premature retirement, thus emphasizing the need for providing deeper insight into the factors that affect treatment outcomes and for offering thorough guidance for the assessment and management of this condition. Taking into consideration the rising prevalence of cLBP and the knowledge gap referring to the overall health benefits of balneotherapy (BT), the aim of this study was to assess the effects of BT on functional status, quality of life, and psychological symptoms in patients with cLBP. **Methods**: Among 220 patients from the database, two groups were retrospectively identified: patients receiving conventional pharmacological therapy (CT) and patients receiving BT. The effectiveness of the treatment was assessed through a visual analog scale for pain intensity, EuroQol (EQ-5D), and the Work Ability Index Questionnaire. In order to provide deeper insight into the quality of life and also factors associated with functional status and mental health affected by BT, we also used the Short Form Health Survey Questionnaire and the Center of Epidemiologic Studies Depression Scale. **Results**: Both age and treatment protocol were found to have a significant impact on all observed parameters, i.e., older patients and those receiving CT tended to report lower overall health and physical functioning. On the other hand, BT was associated with better functionality and disability perception status. **Conclusions:** Understanding the association between individual perceptions of functionality and its emotional and social elements provides a basis for providing comprehensive guidelines and recommendations for cLBP management.

## 1. Introduction

Chronic low back pain (cLBP) represents a serious public problem and is a leading cause of disability worldwide that profoundly impacts both the quality of life of patients and healthcare costs [1]. Data indicate that LBP affected around 619 million people globally in 2020, while the prevalence rate for cLBP is around 10–20%, depending on the region and specific population studied. It is projected that the number of LBP cases will rise to 843 million by the year 2050, along with the overall burden of disability and the associated costs related to this condition [2]. Available treatment modalities include pharmacological approaches based on nonsteroidal anti-inflammatory drugs (NSAIDs) and non-pharmacological strategies involving physiotherapy and physical activities. Although therapy for chronic low back pain can be beneficial, it also has serious disadvantages, such as incomplete efficacy, limited long-term solution, occurrence of side effects, and lack of impact on psychological aspects. Moreover, cost and accessibility represent barriers for the majority of patients, since therapy sessions are expensive and not always covered by health insurance [3,4,5].

Balneotherapy (BT) involves the use of mineral baths or thermal waters and has been used for centuries for different therapeutic purposes. The therapeutic outcome is influenced by the characteristics of the mineral water, the duration and frequency of treatment sessions, the individual response, etc. [2,6]. BT can exert numerous advantages such as pain relief, muscle relaxation, and improved physical function, providing significant relief to individuals suffering from cLBP [6,7]. The non-pharmacological nature of BT underscores its potential as a safer solution compared to conventional treatment protocols, i.e., medications or invasive procedures. It has been considered that the thermal effect, hydrostatic pressure, and mineral content contribute to muscle relaxation and to the alleviation of stress on the spine and joints, thus exerting analgesic effects [2,6,7]. Previous data suggest that BT also exerts positive effects on mental health due to relaxation and sensory experiences that result in stress and anxiety alleviation [8]. Although recent studies have revealed the benefits of BT for chronic conditions such as cLBP, efforts are continuing to be invested in the development of standardized treatment protocols with the aim of optimizing the effectiveness of BT in managing this condition. Moreover, there is a lack of comprehensive data that fully elucidate the connection between BT and its effects on functional status, quality of life, depression, and mental disorders in individuals with cLBP, while most available information pertains to elderly individuals [4,6,7,9]. Therefore, addressing multidimensional aspects of both the physical and psychological effects of BT is essential for supporting healthcare providers by updating guidelines that will contribute to enhanced patient outcomes and quality of life and improve overall patient well-being.

The goal of this study was to assess the effects of BT on functional status, quality of life, and psychological symptoms in patients with cLBP. Additionally, the specific goal was to determine which individual patient characteristics (such as age or gender) might be linked to the effectiveness of BT. These findings will contribute to the updating of clinical practice guidelines and advancing evidence-based practices in cLBP management.

## 2. Materials and Methods

This was an analytical cross-sectional study that included 220 patients with cLBP aged 18–65 years. The examined patients included those admitted to the Community Health Center Kragujevac, Serbia, and hospitalized patients with the aforementioned syndrome at the Special hospital for treatment and rehabilitation “Merkur” in Vrnjačka Banja, Serbia, who underwent a specific balneophysical treatment. The evaluated period was from December 2019 to December 2020. This study was initiated with the approval of the Ethics Committee of Community Health Center Kragujevac (date: 9 June 2014, decision number: 01-2117/1) and Ethics Committee of the Special hospital for treatment and rehabilitation “Merkur” in Vrnjačka Banja (date: 9 June 2014, decision number: 01-5617/6). All procedures were carried out in accordance with the ethical rules and the principles of the Declaration of Helsinki.

All the participants provided written informed consent. Inclusion criteria for all patients were as follows: adults of both genders, presence of back pain for more than 12 weeks, age older than 18 years, voluntary agreement to participate in this study, and confirmed non-specific cLBP. Non-specific cLBP syndrome was confirmed by a physician based on the medical history, clinical manifestations, negative laboratory indicators of inflammation (blood sedimentation, C-reactive protein, and normal urine findings), the presence of pain for more than 12 weeks, and a radiograph of the lumbosacral spine in two directions.

Exclusion factors for both groups were as follows: subjects less than 18 years of age, subjects with cancer and metastases, pregnant women, subjects with inflammatory processes and conditions after surgical intervention due to wrong perception of pain, subjects with spinal canal stenosis, osteoporosis, osteoporotic fractures of the vertebral bodies, spondylitis, sacroilitis, osteomyelitis, subjects with conditions that lead to a reflex pain syndrome (kidney conditions, conditions of the small pelvis and abdominal organs), and people who refused to participate in the research.

The sample size for this study was determined using the statistical software tool G*Power version 3 based on the results of a study in which a therapeutic program similar to the complex impact of a longer BT treatment on the whole organism was applied [10,11]. A *t*-test for two independent samples was used, with an alpha (Type I error rate) of 0.05; the power of this study was 0.8, and the double comparison and ratio of the number of subjects were 1:1. The sample size was 106 per group and after considering a certain part of nonresponses and refusals, the total sample size adjusted for this study was 220 participants.

### 2.1. Study Design

Among 220 patients from the clinic database, two groups were retrospectively identified: conventional therapy (CT) and balneotherapy (BT). The groups were based on a treatment protocol recommended by a specialist in physical medicine. The doctor prescribed CT or decided to subject patients to BT based on their condition, including the duration, severity, and nature of their chronic low back pain, as well as clinical examination involving functional status, pain levels, range of motion, and diagnostic imaging and tests. The decision on whether BT was suitable for each study participant was based on a combination of relevant evidence from the guidelines and protocols and patient-specific considerations.

Patients belonging to the CT group were treated at the Community Center Kragujevac and prescribed drug therapy, which included NSAIDs and/or analgesics, muscle relaxants, and antidepressants. The physicochemical characteristics of the water used for the BT protocol are presented in Table 1. During BT, patients remained standing with their arms immersed up to the neck in a pool containing thermomineral water at a temperature of 29–31 °C. The treatment involved general baths with low radioactivity lasting 20 min that were performed under the supervision of a physiotherapist, who also served as the exercise demonstrator.

Patients receiving BT completed questionnaires on the day of discharge from the Special hospital for treatment and rehabilitation “Merkur” in Vrnjačka Banja, while those who did not receive the BT treatment completed the questionnaire at the same time to ensure that the data were captured under similar conditions.

### 2.2. Evaluation Parameters

The demographic characteristics of the patients, such as age, gender, height (m), weight (kg), body mass index (BMI) (kg/m^2^), condition duration, and number of BT treatments, were recorded.

The EQ-5D 3-stage version was used for assessment of health-related quality of life in patients with cLBP. It comprises two pages of the EQ-5D descriptive system and the EQ-VAS (visual analog scale). The EQ-5D assesses five domains of quality of life (mobility, self-care, daily activities, mood, pain/discomfort) [12]. The EQ-VAS included a thermometer-like scale, where the respondent evaluated their own state of health with grades from 0 (worst quality) to 100 (best). The EuroQol group approved the use of this questionnaire for the purposes of this research under number 20561. Cronbach’s alpha value for the EQ-5D-3L was 0.70.

A VAS scale was used to assess the degree of pain. The patients marked their pain severity on a 100 mm line, ranging from 0 to 100 mm. The distance from the lowest VAS value to the patient’s mark indicated the numerical severity of their pain [12].

The Short Form 36 (SF-36) was used to evaluate quality of life. This scale consists of 36 questions related to health, divided into 8 domains: physical functioning (PF), role—physical (RP), bodily pain (BP), general health (GH), vitality (VT), social functioning (SF), role—emotional (RE), and mental health (MH). Total physical health (PCS—Physical Component Summary) summarizes the PF, RP, BP, and GH dimensions, while total mental health (MCS—Mental Component Summary) is based on the following domains: VT, SF, RE, and MH. The scores of the questions for each status are scored between 0 (worst health condition) and 100 (best health condition). Cronbach’s alpha values for the SF-36 range from 0.70 to 0.90, depending on the specific domain [13,14]. A research license for the use of this standardized questionnaire was filed in the agreement under the number OPTUM#CT193013 OPO68219 OGSR.

The Work Ability Index Questionnaire (WAI), a standardized questionnaire of the Finnish Institute for Occupational Medicine, is used to examine work ability in relation to the requirements of the job or workplace. Cronbach’s alpha values for the WAI questionnaire range from 0.80 to 0.90. The use of this questionnaire is not conditional on obtaining a license [15].

The Center of Epidemiologic Studies Depression Scale (CES-D) was used for assessing depression and to identify groups at risk for depression. It contains 20 items related to the frequency of symptoms during the last seven days. Cronbach’s alpha values for the CES-D questionnaire are in the range of 0.85 to 0.90 [16].

### 2.3. Statistical Analysis

Statistical analyses in this study were carried out by a researcher who was blind to the treatment of each group. The Kolmogorov–Smirnov test was used to determine whether the distribution of the variables was suitable for normal distribution. Numeric variables were compared based on their distribution using either analysis of variance (ANOVA) for normally distributed variables or the Kruskal–Wallis test for those not following a normal distribution. Post hoc analysis utilized the Mann–Whitney U-test and Bonferroni test. The chi-square test was used to compare qualitative data. Regression analysis was used to reveal the association between factors and scores. Statistical significance was set at *p* < 0.05, as determined by SPSS version 20.

## 3. Results

### 3.1. Demographic Characteristics of Participants

A total of 220 patients with chronic non-specific low back pain syndrome were recruited and analyzed in the current study. The characteristics of the study population are shown in Table 2. The age in the CT group was 44.01 ± 11.7 years, while in the BT group, it was 48.95 ± 7.3 years. There was a significant association between the treatment groups in terms of age categories (*p* = 0.00). In patients belonging to the CT group, 50.91% were female and 49.09 were male, while in the BT group, 30.09 were female and 69.09 were male. A statistically significant difference was revealed between the examined groups in terms of gender distribution (*p* = 0.003). Additionally, there was also a statistically significant difference in BMI categories and condition duration between the CT and BT groups (*p* = 0.00).

### 3.2. EQ-5D-3L and SF-36 Questionnaires

To determine whether there was a statistically significant difference in EQ-5D-3L descriptive system categories between the CT and BT groups, the Mann–Whitney U test was performed. There were statistically significant differences between patients receiving BT and CT in terms of mobility and usual activities (*p* < 0.05). On the other hand, there were no statistically significant differences in self-care, pain/discomfort, and anxiety/depression between the CT and BT groups (*p* > 0.05) (Table 3).

The overall health score assessed using the visual analog scale (EQ-VAS) was significantly lower in patients receiving CT (60.96 ± 17.16) compared to BT (68.46 ± 16.93) (*p* < 0.05) (Table 3).

In order to reveal whether there was a statistically significant difference in SF-36 domains and their values between the CT and BT groups, the Mann–Whitney U test was performed. There were statistically significant differences between patients subjected to BT compared to CT in terms of all domains except general health (*p* < 0.05) (Table 3).

**Figure 1:**
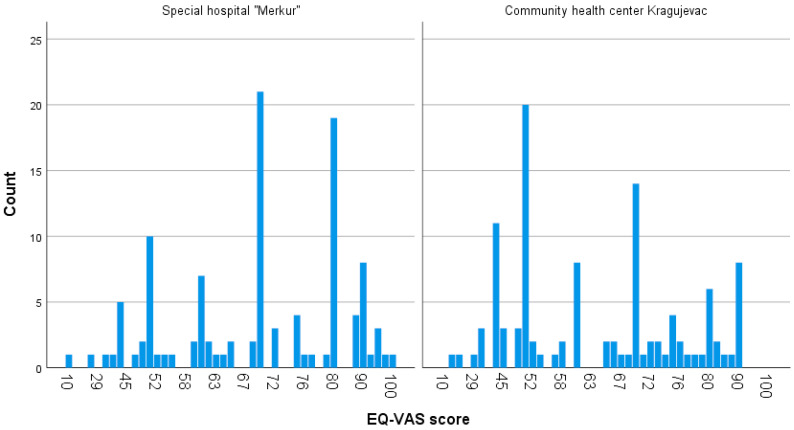
Distribution of EQ-VAS scores (n = 220) among patients receiving CT and BT, where higher values of EQ-VAS suggest improved self-rated health.

In order to reveal whether there is an association between age, gender, BMI categories, condition duration, and number of BT treatments and EQ-5D-3L descriptive system categories, the chi square test was performed. There was no association between gender, BMI groups, condition duration, number of BT treatments, and EQ-5D-3L descriptive system categories (*p* > 0.05), while there was a statistically significant interaction in terms of age categories (≤45 or >45 years) and self-care (*p* = 0.04), usual activities (*p* = 0.03), and anxiety/depression (*p* = 0.038). Distribution of EQ-VAS scores among patients receiving CT and BT is presented in Figure 1.

Associations between the sociodemographic and clinical characteristics of the patients and their EQ VAS and SF 36 (PCS and MCS) scores are shown in Table 4.

The assessment of factors associated with EQ VAS scores revealed statistically significant interactions between patients ≤45 years and those older than 45. Younger patients had significantly better EQ-VAS scores and improved self-rated health (67.21 ± 16.8 vs. 61.88 ± 16.9). Otherwise, there were no statistically significant associations between other parameters and EQ VAS scores (*p* > 0.05) (Table 4).

Associations between factors related to patients and their total physical health (PCS) and total mental health (MCS) scores are presented in Table 4. Statistical analysis revealed significant gender differences in terms of MCS, with higher MCS scores (38.78 ± 7.72) in males than in females (34.97 ± 7.07). Additionally, there was a significant association between condition duration and both PCS and MCS. In brief, higher scores for PCS were present in patients younger than 45 than in those older than 45, i.e., 45.91 ± 7.2 and 42.01 ± 6.9, respectively. Similarly, MCS scores were significantly elevated in patients ≤45 years age (40.9 ± 8.11) in comparison to those older than 45 years (36.92 ± 7.5) (*p* < 0.05). Moreover, there was a significant link between the number of BT sessions and MCS, and increased MCS values were present in patients exposed to more than one BT session.

Multiple linear regression analysis was conducted to identify factors associated with EQ VAS scores, PCS, and MCS in patients with cLBP (Table 5). Age and treatment protocol were significantly negatively associated with EQ VAS and PCS scores, while age, gender, and treatment type were negatively associated with MCS scores (*p* value < 0.05). CT was associated with lower EQ VAS scores, PCS, and MCS compared to BT.

### 3.3. WAI Questionnaire and VAS Score

The average reported work ability as measured by the WAI was moderate, i.e., 35.01 ± 7.67 points, with 20.5% reporting poor, 30% moderate, 34.5% good, and 15% reporting excellent work ability. Patients receiving BT reported statistically significantly higher WAI (36.89 ± 7.8 points) compared to the CT group (33.14 ± 7.36 points) (Table 6).

Results for VAS scores among the patients are shown in Table 5. VAS scores were significantly higher in the CT group of patients (54.7 ± 21.3) compared to the BT group (45.7 ± 23.01).

Associations between sociodemographic and clinical patient characteristics and the WAI are presented in Table 7. The distribution of WAI scores varied significantly between genders and between different age groups, condition durations, and number of BT treatments.

The Mann–Whitney U test showed that patients 45 years old and younger had statistically higher WAI scores than older ones (*p* < 0.05). Additionally, male patients reported significantly higher WAI scores than female ones (36.05 ± 8.02 in males vs. 33.52 ± 6.81 in females) (*p* < 0.05). Higher WAI scores were found in patients who had had low back pain for less than a year than in those whose condition duration was more than a year (36.84 ± 7.13 vs. 34.30 ± 7.78). There was a significant difference in WAI scores between patients who received one and more than one balneotherapy session (34.45 ± 7.62 for one balneotherapy session and 36.20 ± 7.7 for more than one balneotherapy session) (*p* < 0.05).

Additionally, there was a significant association between condition duration, the number of balneotherapy sessions, and VAS scores (*p* < 0.05) (Table 7). Patients suffering from low back pain for longer than 1 year had higher VAS scores (52.44 ± 22.0) compared to those with established diagnosis for less than a year (44.52 ± 23.2). Moreover, patients who received more than one balneotherapy session had significantly lower VAS scores (46.41 ± 21.72) than those subjected to one therapy session (52.02 ± 22.8).

Multiple linear regression analysis revealed that age and treatment protocol were significantly negatively associated with WAI scores, while treatment type was positively associated with VAS, i.e., patients subjected to CT tended to have lower WAI and higher VAS scores (*p* value < 0.05) (Table 8).

**Figure 2:**
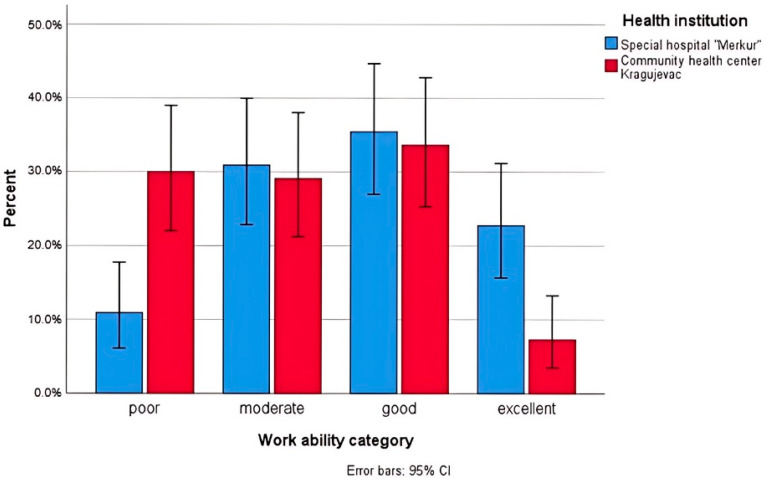
Distribution (%) of Work Ability Index scores according to category (n = 220).

The distribution of the work ability categories between patients treated with conventional protocols and BT is presented in Figure 2.

### 3.4. CES-D Scale

There were statistically significantly lower CES-D scores in patients receiving BT compared to CT, thus indicating improved levels of depression (Table 9).

The distribution of CES-D scores varied significantly between gender and age groups (Table 10). The Mann–Whitney U test showed that patients 45 years old and younger had statistically significantly lower CES-D scores than older ones (*p* < 0.05). Additionally, female patients had significantly higher CES-D scores than male patients (16.7 ± 4.11 in females vs. 12.11 ± 2.9 in males) (*p* < 0.05).

It was observed that age and gender were significantly positively associated with CES-D scores, while treatment protocol was negatively associated with CES-D scores (*p* value < 0.05, Table 11).

## 4. Discussion

In the current study, positive associations between BT and functional state, quality of life, and mental health in cLBP patients at mid-life were observed. This study provides comprehensive evidence of multiple aspects that affect patient’s well-being. The studies available so far have mostly been oriented on the identification of specific impacts on pain, functional state, and disability [6,7]. We provide a comprehensive understanding of how BT can alter physical and mental healthcare indicators, which is a crucial step in advancing evidence-based practice in pain management. The present study is among the few that followed the impact of BT on cLBP, specifically in individuals at mid-life, with demographic characteristics closely resembling those typically seen in BT treatments for cLBP [17].

There were statistically significant differences in age, BMI, and disease duration between the groups, which was expected in this type of research due to the criteria for initiating BT being associated with specific patient characteristics. The higher number of older participants in the BT group can be explained by the fact that, with the aging and increased sensitivity to side effects of patients, doctors recommend BT more often due to its non-invasive nature and perceived lower risk of side effects compared to pharmacological treatments. Moreover, BT is likely to be initiated in patients with higher BMI who have greater musculoskeletal stress, as it can help alleviate joint and muscle issues [18,19].

The EQ-5D-3L descriptive system was used to measure health-related quality of life in patients receiving BT and provide a patient-centered perspective on the benefits of this type of therapy. Our findings suggest that in patients with cLBP, BT and younger age were associated with better mobility and usual activities, which are among the essential components of quality of life and thus directly contribute to patients regaining their independence and functionality [20]. Addressing these core aspects of functional limitations enables patients to experience less pain, perform daily tasks with greater ease, and reduce the necessity for medication reliance. Additionally, higher EQ-VAS scores and lower VAS scores in patients receiving BT compared to CT may indicate that BT has the potential to improve health perception and better psychological well-being. These findings undoubtedly serve as a valuable subjective measure of the contribution of BT, along with other patient characteristics, to overall health in individuals suffering from cLBP.

In addition to the aforementioned questionnaires, we also used the SF-36 to monitor the influence of BT and revealed that BT impacts not only pain but also broader aspects of health-related quality of life. Regression analysis revealed significant improvement in physical functioning and role limitations due to physical health, bodily pain, vitality, and social functioning in patients that received BT, independently of the effects of age and gender. These findings indicate the potential of BT as a promising strategy for cLBP management via its impact on different aspects of a person’s life. Improvements in physical and mental health are important because they enhance the quality of life, reduce disability, and improve treatment adherence [1,2].

Based on our data, we may propose initiating BT earlier to prevent the progression of acute or subacute low back pain into a chronic condition with severe symptoms. However, consulting with a healthcare provider is crucial to determine the most appropriate timing and approach to treatment based on individual circumstances. Multiple linear regression analysis referring to the quality-of-life parameters revealed that as people get older, their EQ VAS and PCS and MCS scores tend to be lower, suggesting a decline in both physical and mental health components with age. Moreover, patients subjected to BT tended to have higher EQ VAS, PCS, and MCS scores and therefore better quality of life. The greater vulnerability of women to most of the monitored aspects of cLBP revealed in this research emphasizes the importance of the implementation of education strategies about the gender differences in cLBP, which will contribute to the prevention and alleviation of the condition’s progression [6,15].

It was previously reported that BT in elderly people with cLBP led to improvements in functionality assessed through VAS scores when implemented in combination with physical therapy [21]. In line with our data, it was also confirmed that BT can be effective in pain reduction and improvement in functionality, fatigue, and sleepiness in patients with knee osteoarthritis [10,22]. Another group of authors revealed that BT reduces pain intensity and functional disability, as well as improving perceived self-efficacy, fears, and beliefs about physical activity and pain [4].

It has not been fully clarified so far how the number of balneotherapy sessions affects VAS scores in cLBP [23]. Generally, it is difficult to identify the exact number of BT sessions required for an improvement in the functional state of patients since the treatment response varies widely among individuals and the specific protocol of BT affects clinical outcomes. The number of sessions recommended for cLBP can vary based on the severity of the pain and it is often determined on a case-by-case basis by healthcare providers [24,25].

The WAI questionnaire was also used for data collection in this research study in order to provide a more detailed understanding of the influence of BT on patients’ ability to work and to predict work disability [15]. We revealed significantly higher WAI scores in patients subjected to BT compared to CT, which typically indicates better perceived work ability and functional capacity. Statistical analysis revealed that both patients subjected to BT and younger patients tended to have higher WAI scores. Additionally, BT, independently of other clinical characteristics, was associated with lower VAS scores. The aforementioned results highlight the advantages of BT for patients in terms of both work ability and pain relief. In fact, these findings reflects how BT can help patients to alleviate condition-induced limitations in daily working tasks and enhance their ability to engage in work-related activities. The significantly higher WAI scores in men with cLBP may likely be explained by variations in pain perception, coping mechanisms, hormonal influences, and functional capacity; moreover, men may be less likely to report pain [26].

Apart from their physical manifestations, which strongly limit the functional state of patients with cLBP, chronic pain conditions are frequently associated with depression. Prolonged pain affects daily life and social interactions, emphasizing the necessity of providing patients with therapy that would also prevent depressive symptoms and improve their mental health [27]. In this context, we analyzed CES-D scores in order to provide a comprehensive insight into the benefits of BT, both to physical and psychological aspects. In our study, a decreased CES-D score after BT reflects improved emotional health, reduced depressive symptoms, enhanced quality of life, and a positive response to BT in cLBP. It underscores the holistic benefits of BT in addressing not only the physical symptoms but also the psychological aspects of chronic pain. Özkuk et al. observed a greater reduction in anxiety following BT and suggested that this could be attributed to the interplay between pain and anxiety [28]. It has been proposed that BT exerts its benefits through stress reduction alteration in salivary cortisol levels in both healthy individuals and in individuals with cLBP [29]. Moreover, the hydrostatic pressure and warmth of the water may promote endorphin release, thus contributing to pain relief, which is in accordance with our findings [30]. We noticed significantly higher CES-D scores in women, which appears to be logical due to the already proven increased vulnerability to mood changes and depressive symptoms in women [31].

Both older age and undergoing CT were significantly associated with poorer reports of overall health and physical functioning. In light of this, this research study provides valuable information for increasing the awareness of healthcare providers regarding the protective effects of BT on pain management and improvement in functional status and patients’ quality of life. Taking into consideration the incidence of depressive symptoms in cLBP patients and the improvement in mental health achieved by BT, evidence-based practice should highlight BT along with psychosocial support and resources to address the emotional impact of chronic pain. Moreover, these data should encourage employers to implement strategies such as ergonomic assessments, adjustments to workstations, and education on proper lifting techniques to reduce the risk of developing chronic pain.

## 5. Conclusions

Our results indicate that BT is associated with reduced pain, improved functionality, and enhanced disability perception, supporting its effectiveness in managing cLBP through rehabilitation. Understanding the physical and mental healthcare indicators and patients’ health perception affected by BT is essential for optimizing patient care and adjusting treatment frequency and duration or for combining therapies to optimize outcomes. The limitations of our study include focusing on the immediate benefits of BT, without accounting for the body’s recovery and adaptation, which may reveal more long-term effects of BT over time. Incorporating follow-up assessments and an examination of long-term outcomes can offer a more complete understanding of the treatment’s benefits and its sustainability over time.

## Figures and Tables

**Table 1:** Physical and chemical features of the water used for BT.

Components	Water
Capped depth (in meters)	300 m
Generosity (L/s)	6.8
Water temperature (°C)	36.6
Total mineralization (g/L)	2.76
Type of water	NaHCO_3_
pH value	6.70
Water hardness/general	28.84
Electrical conductivity (μS/cm)	2.780

Generosity—the concentration of overall mineral content in liters per second, indicating the flow rate of the spa water from its source; capped depth—the depth at which the spa water was extracted.

**Table 2:** Characteristics of patients in the examined groups: conventional pharmacological therapy (CT) and balneotherapy (BT) (n = 220).

Variable	CT (n = 110)	BT (n = 110)	Statistics
**Age (mean + SD)**	44.01 ± 11.7	48.95 ± 7.3	χ2 = 13.279 *p* = 0.00
≤45 years	53	27	
>45 years	57	83	
**BMI (kg/m^2^)**			χ2 = 16.67 *p* = 0.00
<25	60	31	
25–29	37	52	
≥30	13	27	
**Condition duration**			χ2 = 12.936 *p* = 0.00
≤1 year	43	19	
>1 years	67	91	
**Gender (%)**			χ2 = 9.101 *p* = 0.003
female	50.91	30.09	
male	49.09	69.09	

The chi-square test of independence was used for comparing the distribution of categorical variables between the CT and BT groups.

**Table 3:** EQ-5D-3L descriptive system, EQ VAS scale, and SF-36 domains in the examined groups: conventional pharmacological therapy (CT) and balneotherapy (BT) (n = 220).

ITEMS	CT (Mean ± SD)	BT (Mean ± SD)	*p* Value
**EQ-5D-3L Descriptive System**			
Mobility	1.63 ± 0.05	1.45 ± 0.49	<0.05
Self-Care	1.36 ± 0.48	1.25 ± 0.43	>0.05
Usual Activities	1.66 ± 0.58	1.46 ± 0.5	<0.05
Pain/Discomfort	2.02 ± 0.45	1.9 ± 0.4	>0.05
Anxiety/Depression	1.88 ± 0.61	1.73 ± 0.60	>0.05
**EQ VAS score**	60.96 ± 17.16	68.46 ± 16.93	<0.05
**SF-36 domains**			
PF	61.41 ± 7.8	68.48 ± 7.2	<0.05
RP	29.06 ± 5.8	32.3 ± 5.37	<0.05
BP	36.9 ± 4.3	43.3 ± 67	<0.05
GH	42.7 ± 7.01	44.6 ± 4.6	>0.05
VT	45.2 ± 7.11	51.6 ± 9.02	<0.05
SF	39.6 ± 6.25	46.18 ± 5.45	<0.05
RE	23.88 ± 3.8	20.47 ± 3.7	<0.05
MH	41.4 ± 8.8	48.18 ± 9.3	<0.05
PCS	41.4 ± 6.8	47.35 ± 7.2	<0.05
MCS	34.55 ± 7.07	41.89 ± 7.3	<0.05

Higher scores for EQ-5D-3L subscales (mobility, self-care, usual activities, pain/discomfort, and anxiety/depression) indicate greater impairment in health-related quality of life, while higher values of EQ-VAS score indicate improved self-rated health. Mann–Whitney U test was used to compare continuous variables between CT and BT groups. PF—physical functioning, RP—role—physical, BP—bodily pain, GH—general health, VT—vitality, SF—social functioning, RE—role—emotional, and MH—mental health.

**Table 4:** Association between factors and EQ VAS and PCS and MCS scores.

	EQ VAS		PCS		MCS	
	(Mean ± SD)	*p* Values	(Mean ± SD)	*p* Values	(Mean ± SD)	*p* Values
Age						
≤45 years	61.88 ± 16.9	<0.05	44.16 ± 7.5	>0.05	38.4 ± 6.9	>0.05
>45 years	67.21 ± 16.8		42.21 ± 6.8		36.5 ± 8.01	
Gender						
male	65.30 ± 16.9	>0.05	43.3 ± 7.4	>0.05	38.78 ± 7.72	<0.05
female	61.68 ± 17.13		42.2 ± 6.7		34.97 ± 7.07	
BMI		>0.05		>0.05		>0.05
<25	63.49 ± 17.95		42.9 ± 7.02		36.60 ± 7.83	
25–29	63.25 ± 11.34		42.5 ± 7.6		38.12 ± 7.56	
≥30	65.82 ± 12.01		43.72 ± 6.4		36.64 ± 7.8	
Condition duration						
≤1 year	65.10 ± 19.1	>0.05	45.91 ± 7.2	<0.05	40.9 ± 8.11	<0.05
>1 year	63.32 ± 16.2		42.01 ± 6.9		36.92 ± 7.5	
Number of BT treatments						
one	65.63 ± 15.54	>0.05	43.03 ± 7.31	>0.05	36.24 ± 7.3	<0.05
more than one	62.95 ± 17.7		42.8 ± 7.12		39.2 ± 8.09	

Mann–Whitney U test was used to compare continuous variables between two groups, while ANOVA was used to compares the means of continuous variables among three or more independent groups.

**Table 5:** Multiple linear regression analysis of the association of patients’ characteristics and treatment protocol with EQ-5D scores, PCS, and MCS.

	EQ VAS Score			PCS			MCS		
	*p* Value	Beta	95% CI	*p* Value	Beta	95% CI	*p* Value	Beta	95% CI
Age	0.004	−0.199	−12.03, −2.32	0.006	−0.19	−4.84, −0.81	0.005	−0.18	−4.94, −0.90
Gender	0.31	−0.070	−7.26, −2.32	0.84	−0.014	−2.19, 1.78	0.007	−0.176	−4.73, −0.76
BMI	0.98	−0.002	−3.27, −3.2	0.85	−0.013	−1.47, 1.22	0.17	−0.09	−2.27, 0.41
TP	0.00	−0.251	−13.52, −3.92	0.001	−0.248	−5.54, −1.54	0.00	−0.382	−7.846, −3.85

TP—treatment protocol (CT or BT); PCS—total physical health; MCS—total mental health. Multiple linear regression analysis was used to understand the relationship between EQ VAS scores, PCS, and MCS and age, gender, BMI, and TP (BT was marked as standard).

**Table 6:** WAI and VAS scores in the examined groups: conventional pharmacological therapy (CT) and balneotherapy (BT) (*n* = 220).

ITEMS	CT (Mean ± SD)	BT (Mean ± SD)	*p* Value
WAI	33.14 ± 7.36	36.89 ± 7.8	<0.05
VAS	54.7 ± 21.3	45.7 ± 23.01	<0.05

Mann–Whitney U test was used to compare continuous variables between CT and BT groups.

**Table 7:** Association between factors and WAI and VAS.

	WAI		VAS	
	(Mean ± SD)	*p* Values	(Mean ± SD)	*p* Values
Age				
≤45 years	36.8 ± 7.2	<0.05	48.6 ± 24.0	>0.05
>45 years	33.94 ± 7.7		51.14 ± 21.8	
Gender				
male	36.05 ± 8.02	<0.05	48.04 ± 23.7	>0.05
female	33.52 ± 6.91		53.34 ± 20.53	
BMI		>0.05		>0.05
<25	35 ± 7.16		44.2 ± 22.22	
25–29	35.03 ± 8.02		50.90 ± 22.3	
≥30	35 ± 8.2		52.13 ± 23.4	
Condition duration				
≤1 year	36.84 ± 7.13	<0.05	44.52 ± 23.2	<0.05
>1 year	34.30 ± 7.78		52.44 ± 22.0	
Number of BT treatments				
one	34.45 ± 7.62	<0.05	52.02 ± 22.8	<0.05
more than one	36.20 ± 7.7		46.41 ± 21.72	

Higher WAI indicates better work ability, while higher VAS score indicates greater pain intensity. Mann–Whitney U test was used to compare continuous variables between two groups, while ANOVA was used to compare the means of continuous variables among three or more independent groups.

**Table 8:** Multiple linear regression analysis of the association of patients’ characteristics and treatment protocol with WAI and VAS scores.

	WAI			VAS		
	*p* Value	Beta	95% CI	*p* Value	Beta	95% CI
Age	0.00	−0.24	−5.93, −1.75	0.11	0.110	−1.26, 11.5
Gender	0.14	−0.10	−3.62, 0.49	0.47	0.051	−3.98, 8.65
BMI	0.33	−0.06	−2.08, 0.70	0.35	−0.066	−6.28, 2.27
TP	**0.00**	−0.31	−6.68, −2.55	**0.006**	0.199	2.61, 15.28

TP—treatment protocol (CT or BT). Multiple linear regression analysis was used to understand the relationship between WAI and VAS scores and age, gender, BMI, and TP (BT was marked as standard).

**Table 9:** CES-D scale in the examined groups: conventional pharmacological therapy (CT) and balneotherapy (BT) (n = 220).

ITEMS	CT	BT	*p* Value
	Mean ± SD		
CES-D	17.27 ± 2.3	11.08 ± 3.28	<0.05

Mann–Whitney U test was used to compare continuous variables between CT and BT groups.

**Table 10:** Association between factors and CES-D scale.

	CES-D Score	
	(Mean ± SD)	*p* Values
Age		
≤45 years	10.6 ± 9.02	<0.05
>45 years	14.68 ± 10.11	
Gender		
male	12.11 ± 2.9	<0.05
female	16.7 ± 4.11	
BMI		>0.05
<25	15.78 ± 10.07	
25–29	14.18 ± 10.34	
≥30	13.27 ± 10.2	
Condition duration		
≤1 year	13.97 ± 11.5	>0.05
>1 years	14.96 ± 9.54	
Number of BT treatments		
one	13.31 ± 10.02	>0.05
more than one	15.33 ± 10.12	

**Table 11:** Multiple linear regression analysis of the association of patients’ characteristics and treatment protocol with CES-D score.

	CES-D		
	*p* Value	Beta	95% CI
Age	0.00	0.237	2.27, 7.68
Gender	0.005	0.186	1.14, 6.48
BMI	0.835	0.014	−1.99, 1.61
TP	0.000	−0.274	2.85, 8.20

TP—treatment protocol (CT or BT). Multiple linear regression analysis was used to understand the relationship between CES-D score and age, gender (male was marked as standard), BMI, and TP (BT was marked as standard).

## Data Availability

The data presented in this study are available on request from the corresponding author.
